# Terpinen-4-ol suppresses proliferation and motility of cutaneous squamous cell carcinoma cells by enhancing calpain-2 expression

**DOI:** 10.32604/or.2024.050661

**Published:** 2025-02-28

**Authors:** DONGYUN RONG, YUSHEN SU, ZHIRUI ZENG, YAN YANG, HONGUAN LU, YU CAO

**Affiliations:** 1 Clinical Medical School, Guizhou Medical University, Guiyang, 550025, China; 2 Public Health School, Guizhou Medical University, Guiyang, 550025, China; 3 School of Basic Medicine, Guizhou Medical University, Guiyang, 550025, China; 4 Department of Internal Medicine, The Third Affiliated Hospital of Guizhou Medical University, Duyun, 558000, China

**Keywords:** Cutaneous squamous cell carcinoma (cSCC), Terpinen-4-ol (T4O), Calpain-2 (CAPN2), Mouse xenograft, Apoptosis

## Abstract

**Background:**

Terpinen-4-ol (T4O), a key constituent of tea tree essential oil and various aromatic plants, has shown promising antiproliferative and pro-apoptotic effects in melanoma and other cancer types. However, its efficacy against cutaneous squamous cell carcinoma (cSCC) remains unclear. Thus, in this study, we investigated the *in vivo* and *in vitro* effects of T4O on cSCC cell lines and preliminarily explored its impacting pathways.

**Methods:**

Using CCK8 and assay colony formation, we assessed the viability of cSCC A431, SCL-1, and COLO-16 cells treated with T40 at varying concentrations (0, 1, 2, and 4 μM). Flow cytometry was employed to evaluate T4O’s effect on cSCC cell’s cycle progression and apoptosis induction. Additionally, western blotting was utilized to examine the expression intensities of N-cadherin and E-cadherin, two indicative markers of the epithelial-mesenchymal transition (EMT) pathway. T4O’s *in vivo* effect on inhibiting tumor progression was evaluated on an established xenograft tumor model. Then, the molecular mechanisms of T4O’s antitumor effect were explored by an integrated genome-wide transcriptomics and proteomics study on cSCC A431c cells. Finally, calpain-2’s potential mediator role in T4O’s anti-tumor mechanism was investigated in calpain-2 knockdown cell lines prepared via siRNA transfection.

**Result:**

It’s demonstrated that T4O treatment inhibited cSCC proliferation, clonogenicity, migration, and invasion while inducing apoptosis and suppressing the EMT pathway. T4O administration also inhibited cSCC tumorigenesis in the xenograft tumor model. RNA-sequencing and iTRAQ analysis detected significant upregulation of calpain-2 expression in T4O-treated cSCC cells. Western blotting confirmed that T4O significantly increased calpain-2 expression and promoted proteolytic cleavage of β-catenin and caspase-12, two calpain-2 target proteins. Importantly, siRNA-mediated calpain-2 knockdown relieved T4O’s suppressive effect on cSCC cell proliferation and motility. Mechanistically, T4O upregulates calpain-2 expression and promotes the cleavage of β-catenin and caspase-12, with siRNA-mediated calpain-2 knockdown mitigating T4O’s suppressive effects.

**Conclusion:**

These findings suggest that T4O’s antitumor activity in cSCC is mediated through the upregulation of calpain-2 expression and subsequent modulation of β-catenin and caspase-12.

## Introduction

Cutaneous squamous cell carcinoma (cSCC) represents about 20% of nonmelanoma skin cancer cases worldwide and is the second most common malignant tumor among whites [1]. Mainly caused by prolonged ultraviolet (UV) radiation exposure, cSCC occurs about 3–4 times more in men than in women, with its incidence increasing with age. Despite the high cure rate yield by surgical removal of small (<2 cm) cSCC tumors, tumor recurrence often occurs with larger lesions, among which 4% of the cSCC tumors would metastasize after therapy [2,3]. Therefore, novel drugs treating cSCC are urgently needed for the significantly worsened symptoms of tumor recurrence and metastasis.

Terpinen-4-ol (T4O), a natural form of monoterpene, is the main bioactive component of essential tea tree oil which also exists in many aromatic plants [4]. T4O exhibits antimicrobial, antitumoral, anti-aging, and anti-inflammatory effects in previous research by reducing the accumulation of leukocytes [5,6]. Di Martile et al.’s study showed that T4O enhanced apoptosis in melanoma cells treated with dabrafenib and/or trametinib [7]. Several studies also reported T4O’s antiproliferative and pro-apoptotic effects in colorectal, pancreatic, prostate, gastric, and non-small cell lung cancer [8]. Till now, however, the effects of T4O on cSCC have not been clarified.

As a group of calcium-dependent neutral cysteine proteases, calpains are critically involved in the regulation of cell death mechanisms [9,10]. Fifteen calpain family members have been identified in mammals; some of them are ubiquitously expressed, while the others show tissue specificity [11]. Beyond their roles in apoptosis regulation, various calpains participate in diverse cellular functions, including cytoskeletal remodeling, synaptic plasticity, cell migration, and autophagy modulation [12–15]. Notably, Calpain-1 (CAPN1) and calpain-2 (CAPN2) are the most extensively studied Calpain members. Calpain-1 is activated by physiological calcium levels, while calpain-2 is activated only after supraphysiological calcium elevations [16].

Variations in calpain expression and function are associated with severe pathological changes such as lethality, muscular dystrophy, lissencephaly, and tumorigenesis [17]. Noteworthy, accumulating evidence has revealed that calpains might be involved in dual-direction regulations in cancer cell growth, migration, and apoptosis. While a slight increase in calpain-2 expression might promote cancer progression through cleaving different tumor suppressors, a more significant increase in calpain-2 overexpression would induce apoptosis via cleaving a series of proteins necessary for cell survival [18]. Therefore, strategies aimed at increasing calpain-2’s gene expression and activity stand potential for cancer therapy. In this study, we explored T4O’s anti-tumor activity on cSCC both *in vitro* and *in vivo* and unmasked the critical role of calpain-2 as a central effector of T4O’s antitumor actions.

## Materials and Methods

### Cell culture and siRNA transfection

WS1 skin fibroblasts and the CSCC cell lines A431, SCL-1, and COLO-16 were procured from ATCC (American Type Culture Collection, Manassas, VA, USA). All cells were cultured in DMEM (Gibco, New York, NY, USA) with 10% fetal bovine serum (FBS; BI, Kibbutz Beit-Haemek, Israel) at 37°C in a 5% CO_2_ atmosphere. T4O and colchicine were bought from MCE (Wuhan, China). Negative control and calpain-2-targeting (si-calpain2) siRNAs were bought from iGenebio (Beijing, China). The sequence of negative control siRNA was 5′-GCGGTCAGATACCTTCATCAA-3′, and the sequence of si-calpain2 was 5′-AATTCTCCGAACGTCTCACGT-3′. Following the manufacturer’s protocol, siRNAs transfections were performed using Lipofectamine 2000 (Thermo Fisher Scientific, Waltham, MA, USA).

### Cell viability assay

WS1, A431, SCL-1, or COLO-16 cells were plated at a density of 4 × 10^3^ cells/well in 96-well plates and treated with 0 (isovolumetric DMSO), 1, 2, or 4 μM T4O. After 24 and 48 h of incubation, 100 μL DMEM containing 10 μL CCK-8 reagent (Dojindo, Mashiki, Japan) was added to each well, followed by a 2 h incubation at 37°C. The absorbance was measured at 450 nm using a microplate reader.

### Colony formation assay

cSCC cells were plated at a density of 1.5 × 10^3^/well in six-well plates and treated with varying concentrations (0, 1, 2, or 4 μM) of T4O. After 15 days of incubation, the cells were fixed with 4% paraformaldehyde (PFA) for 20 min and stained with 0.1% crystal violet. Colony formation was quantified under a microscope.

### Cell cycle and apoptosis analyses

Cell cycle distributions and apoptosis rates were determined using flow cytometry. An analysis of cell cycle was conducted using a Kit for Cell Cycle Assays (KeyGen, Nanjing, Jiangsu, China). For cell cycle analysis, cSCC cells were plated in six-well plates and synchronized by culturing in FBS-free DMEM. Then, the cells were treated with 0, 1, 2, or 4 μM T4O for 48 h, fixed in 70% ethanol overnight at −20°C, stained with propidium iodide for 30 min, and analyzed. For the apoptosis analysis, cSCC cells were plated into six-well plates and treated with 0, 1, 2, or 4 μM T4O for 24 h. Then the cells were washed three times with PBS, stained with propidium iodide and Annexin V-FITC, and analyzed. Flow cytometry was performed using a DxFLEX flow cytometer (Beckman, CA, USA), and data were analyzed using the FlowJo software [19].

### Western blotting

Western blotting was conducted following the procedure described in a previous study. In brief, the total proteins were extracted from cultured cells by using RIPA lysis buffer with 1% PMSF, and respective concentrations were determined using the Bicinchoninic acid assay (BCA) method (Solarbio, Beijing, China). Protein samples were run on SDS-PAGE gels (Meilun Bio, Dalian, China) and transferred to PVDF membranes (Thermo Scientific, Waltham, MA, USA). After blocking in skim milk powder (Beyotime Biotechnology, Suzhou, China), Using primary antibodies, the membranes were incubated at 4°C for 24 h (purchased from Proteintech, Wuhan, China): Cyclin-D1 (1:1000), CDK2 (1:1000), Bax (1:1000), Bcl2 (1:1000), N-cadherin (1:1000), E-cadherin (1:1000), calpain-2 (1:1000), β-catenin (1:1000), Caspase-12 (1:1000), and β-actin (ACTB; 1:1000). After three washes in Tris-buffered saline containing 0.1% Tween-20, suitable secondary antibodies were applied. A chemiluminescent signal was detected on immunoblots. To determine the relative expression levels of proteins, Actin was used as a control.

### Wound healing assay

cSCC and WS1 cells (5 × 10^5^/well) were seeded into six-well plates. When cell confluence reached ~95%, 0.1 μM colchicine was added and incubated for 12 h. Subsequently, wounds were created in the cell monolayers using a 200 μL pipette tip. After two washes in PBS to remove floating cells, media were replaced by fresh culture medium containing 0, 1, 2, or 4 μM T4O. An optical microscope was used to monitor and record wound closure over a 24-h period.

### Transwell invasion assay

cSCC cells (2 × 10^4^/well) were resuspended in 400 μL of FBS-free DMEM and added to the upper chambers of transwell inserts (0.8 μM pore size; Corning, New York, NY, USA) pre-coated with Matrigel (ThermoFisher Scientific, Waltham, MA, USA). The lower chambers were filled with 600 μL of DMEM with 10% FBS, and T40 (0, 1, 2, or 4 μM) combined with 0.1 μM colchicine, was added to cSCC cells. After 24 h, the invading cells attached to the lower surface of the membranes were fixed and stained with 0.5% crystal violet. The number of cells in five random microscopy fields was counted and averaged.

### Immunofluorescence

cSCC cells were seeded into confocal imaging dishes and treated with either DMSO (control) or 4 μM T4O for 24 h. Then, the cells were washed three times with PBS, fixed with 4% PFA, and permeabilized with 0.2% Triton X-100. After blocking (5% BSA), the cells were incubated with primary antibodies against either N-cadherin (1:200; Proteintech, Wuhan, China) or E-cadherin (1:200; Proteintech, Wuhan, China) overnight at 4°C. Cells were then washed three times in PBS and incubated with FITC labeled-secondary antibodies for 2 h. After DAPI counterstaining, cell nuclei images were captured using a fluorescence microscope at 488 nm.

### Tumor xenograft mouse model

The animal experiments were approved by the Animal Ethics Committee of Guizhou Medical University (Approval number: 2000087). Female BALB/c nude mice (4–6 weeks old) were obtained from the Animal Center of Guizhou Medical University. The nude mice were fed in specific pathogen-free (SPF) conditions at a temperature of 20–26°C, relative humidity of 40%–70%, and light cycle of 12/12 h. 5 × 10^6^ A431 cells, resuspended in 100 μL PBS, were subcutaneously injected into the right axillae of the mice. After the tumor sizes reached 40–60 mm^3^ after approximately 12 days, the mice were randomized into Control (DMSO) and T4O treatment groups (n = 5 for each group). Mice in the T4O group received intraperitoneal injections with 40 mg/kg T4O every 3 days, while mice in the control group received similar volumes of DMSO. Tumor volume was measured every 3 days. All mice were euthanized on day 27 post-treatment, and tumors were extracted for downstream analyses.

### Immunohistochemistry

We fixed tumor tissues in 4% PFA for 6 h, dehydrated them, embedded them in paraffin, and sliced them into 3-mm thick sections. The sections were deparaffinized and rehydrated using xylene and graded alcohols, followed by antigen retrieval in sodium citrate buffer. After washing with PBS, endogenous peroxidase activity was blocked using 3% H_2_O_2_, and nonspecific binding was prevented with 5% BSA (Thermo Scientific, Waltham, MA, USA). Specimens were incubated with primary antibodies anti-PCNA (1:100; Proteintech, Wuhan, China) and anti-Ki67 (1:100; Proteintech, Wuhan, China) antibodies for 24 h at 4°C. After washing, the sections were incubated with HRP-conjugated goat anti-rabbit secondary antibodies (ABclonal, Wuhan, China) for 2 h. The sections were next incubated with DAB reagent (Beyotime Biotechnology, Suzhou, China), counterstained with hematoxylin, and visualized under transmitted light microscopy.

### RNA sequencing

A431 cells were co-incubated with either isovolumetric DMSO (control) or 2 μM T4O (n = 3 cell culture replicates per group) for 24 h. Total RNA was extracted using TRIzol, treated with DNase, and mRNA was purified using oligo(dT)-coated magnetic beads. The mRNA was fragmented using a fragmentation buffer, followed by the synthesis of first-strand cDNA using random hexamer primers. Double-stranded cDNA was then synthesized, purified, end-repaired, and amplified by PCR. An Agilent 2100 Bioanalyzer was used for quality control of the library thus generated, and an Illumina HiSeqTM 2500 instrument was used for sequencing. Clean reads were aligned using HISAT2 for expression quantification. The data was then analyzed using the “EdgeR” R package (R version 4.0.2). Differentially expressed genes were identified using a cutoff of Log_2_FC > 1 and adjusted *p*-value < 0.05.

### Quantitative proteomics (iTRAQ) analysis

A431 cells were co-incubated in triplicate with either DMSO or T4O (2 μM), collected, and ground in liquid nitrogen. Protein expression profiles were detected by iTRAQ [20] by Hangzhou Lianchuan Biotechnology Co., Ltd. (http://www.lc-bio.com/, accessed on 20 April 2024). Briefly, proteins were digested by trypsin, and iTRAQ reagent was used to label the tryptic peptides. LC-MS/MS was then used for protein identification and quantification. Data analysis was conducted using R software, and proteins with *p* < 0.05 combined with Log_2_FC > 1.25 were considered differentially expressed.

### Data analysis

Data were analyzed using SPSS software (version 22.0; IBM Corp., Armonk, New York, NY, USA). Differences between multiple groups were evaluated using Analysis of variance (ANOVA) analysis with Bonferroni’s post hoc test, while differences between two groups were analyzed using Student’s *t*-tests. A significance level of *p* < 0.05 was considered statistically significant.

## Results

### T4O suppresses proliferation and induces apoptosis in cultured cSCC cells

**Figure 1:**
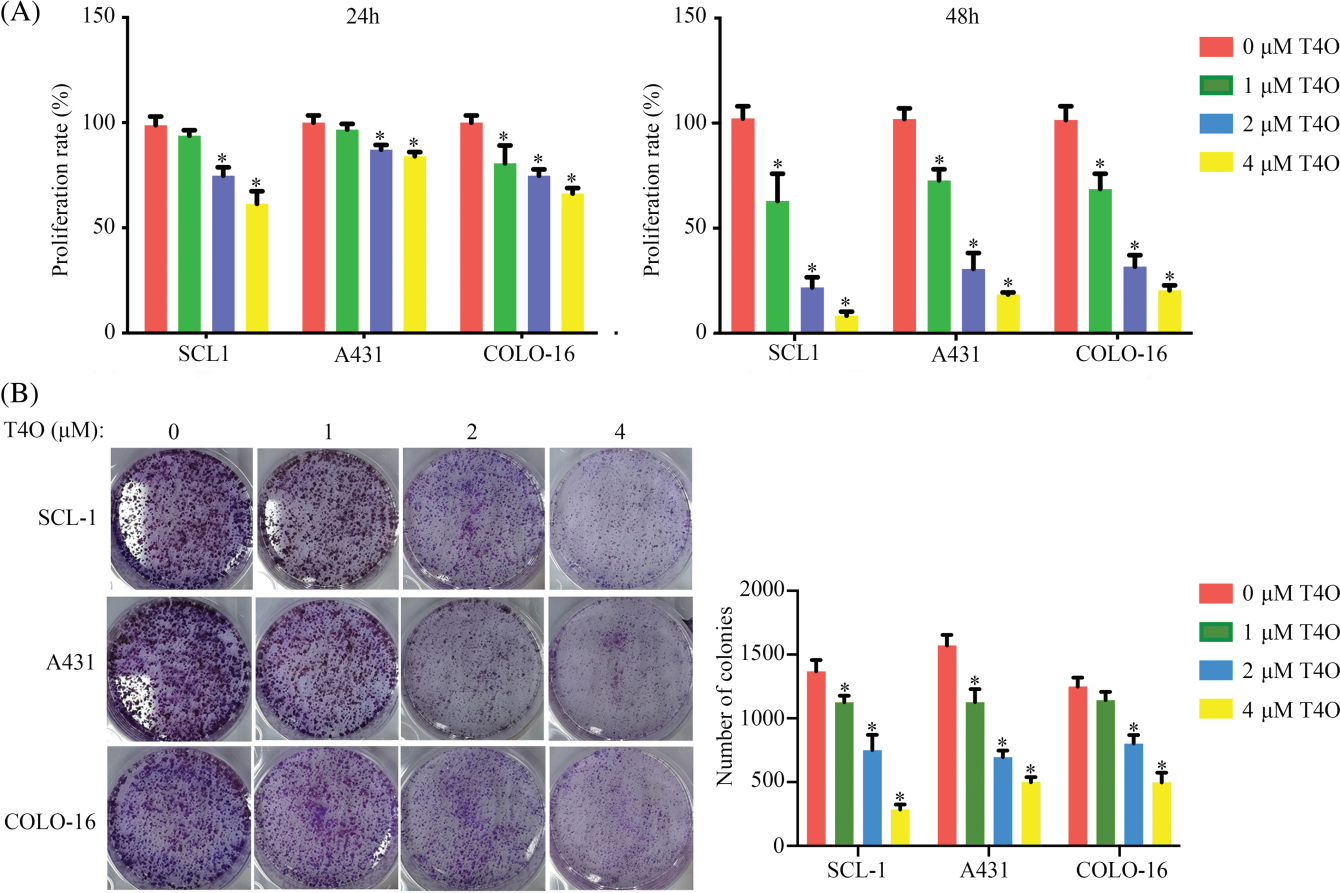
T4O inhibits cSCC cell proliferation *in vitro*. (A) The CCK-8 proliferation assay results of A431, SCL-1, and COLO-16 cells when treated with different concentrations of T4O (0, 1, 2, and 4 μM). (B) The colony formation assay result of cSCC cells when treated with different concentrations of T4O. *p < 0.05.

**Figure A1:**
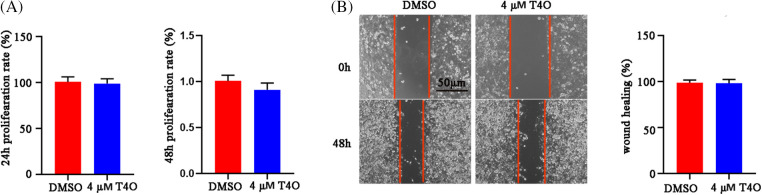
T4O had no effect on proliferation (A) and migration (B) of normal WS1 skin fibroblasts.

T4O’s antiproliferative activity cSCC was assessed in A431, SCL-1, and COLO-16 cell lines treated with varying concentrations (0, 1, 2, or 4 μM) of T4O, as determined by CCK-8 assays. The results showed that T4O significantly reduced the proliferation rate of all three cell lines at both 24 and 48 h after treatment initiation (Fig. 1A). In contrast, we found that the alternation in the proliferation of normal WS1 skin fibroblasts (Fig. A1A) was non-significant after T4O treatment. Next, T4O’s effect on the clonogenic capacity of cSCC cells was investigated by colony formation assays, revealing a decrease in the number of colonies formed by T4O-treated cells compared to that of control cells incubated with DMSO (Fig. 1B).

**Figure 2:**
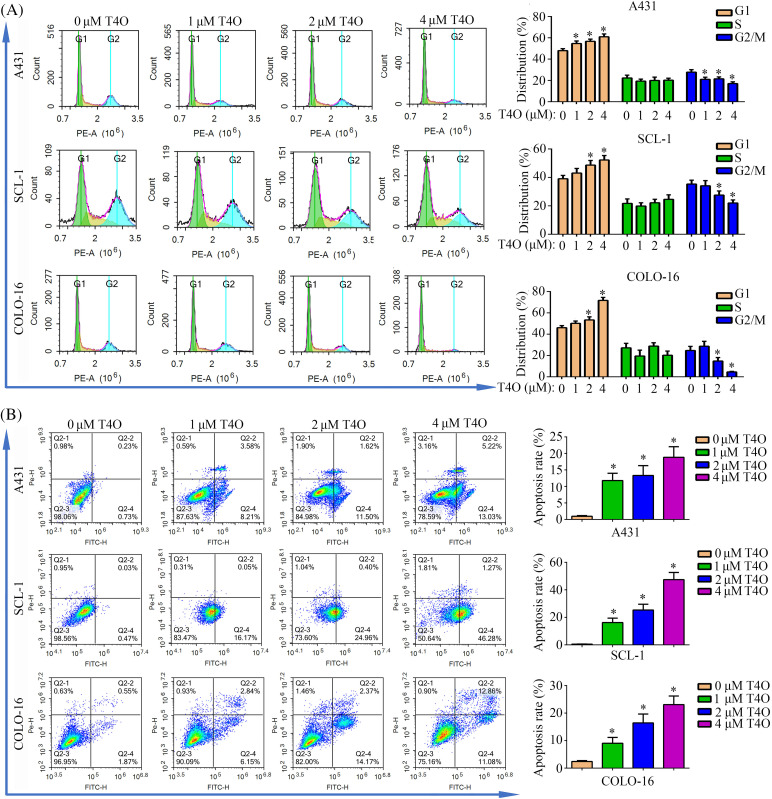
T4O induces G1-phase arrest and apoptosis in cSCC cells *in vitro*. Flow cytometry was used to evaluate cell cycle distribution (A) and apoptosis (B) in A431, SCL-1, and COLO-16 cells treated with different concentrations of T4O. *p < 0.05.

**Figure 3:**
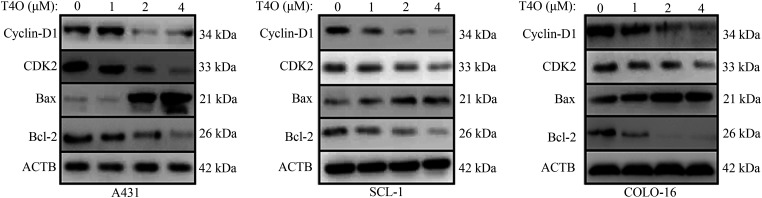
Western blot analysis of cyclin D1, CDK2, Bax, and Bcl-2 expression in cSCC cells. Western blotting was used to explore the expression of cyclin-D1, CDK2, Bax, and Bcl-2 in A431, SCL-1, and COLO-16 cells exposed to different concentrations (0, 1, 2, and 4 μM) of T4O. Western blotting quantification data for A431, SCL-1, and COLO-16 cells.

To further assess T4O’s effect on cSCC cell progression and survival, we evaluated the cell cycle staging and detected apoptosis using flow cytometry. The results indicated G1-phase arrest and a notable reduction in the proportion of cells in G2/M phase following T4O treatment in A431, SCL-1, and COLO-16 cells (Fig. 2A). In turn, the apoptosis rate of A431, SCL-1, and COLO-16 cells was significantly increased after T4O exposure (Fig. 2B). Consistent with these findings, western blotting demonstrated a decrease in the expression of Bcl-2 (an anti-apoptotic marker), cyclin-D1 (a G1 checkpoint protein), and CDK2 (a G1/S transition checkpoint protein), along with an increase in Bax expression (a pro-apoptotic marker) in cSCC cell lines treated with T4O (Fig. 3). These data indicate that T4O suppresses cSCC proliferation through promoting G1-phase arrest and inducing apoptosis.

### T4O inhibits cell motility and epithelial-mesenchymal transition in cSCC cells

**Figure 4:**
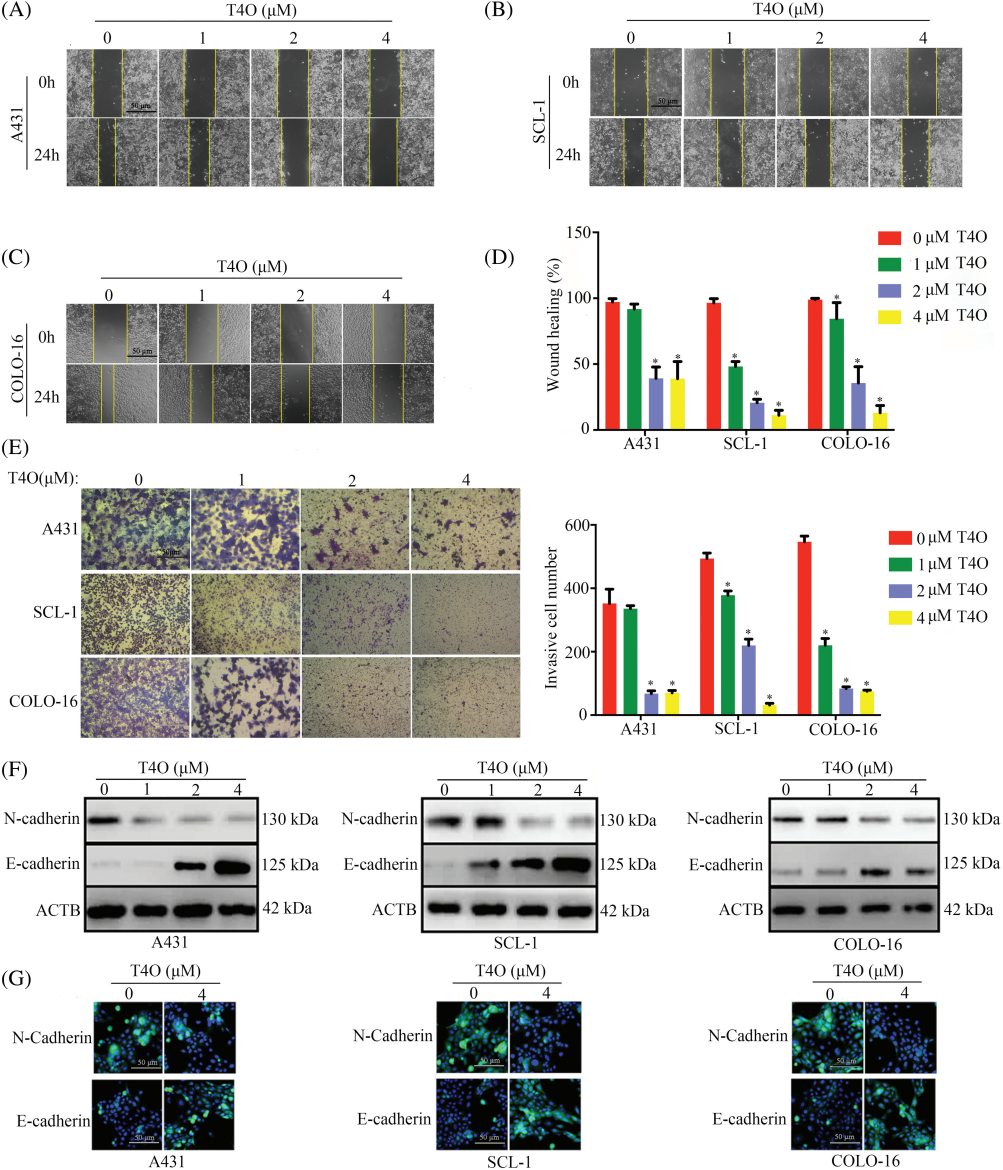
T4O suppresses cSCC cell migration and invasion. (A–D) Wound healing assays were used to assess the effect of T4O on the migration capacity of cSCC cells. (E) Matrigel-Transwell assays were used to evaluate the effect of T4O on the invasive potential of cSCC cells. (F) Western blotting detection of E-cadherin and N-cadherin expression in T4O-treated cSCC cells. (G) Immunofluorescent detection of the expression of E-cadherin and N-cadherin in T4O-treated cSCC cells. *p < 0.05.

To assess the impact of T4O on migration and invasion of cSCC cells, wound healing and Matrigel-Transwell assays were conducted. The results indicated that after T4O treatment migration capacity was drastically reduced in A431, SCL-1, and COLO-16 cells (Fig. 4A–D). In contrast, T4O treatment did not affect wound closure rates in normal WS1 fibroblasts (Fig. A1B). Additionally, Matrigel-Transwell assays showed a significant suppression in the invasive ability of cSCC cells upon T4O treatment (Fig. 4E).

Epithelial-mesenchymal transition (EMT) plays a crucial role in the metastatic dissemination of cSCC [21]. To explore whether T4O inhibits cSCC migration and invasion by blocking EMT, western blot analysis was performed to assess the expression of EMT markers N-cadherin (mesenchymal phenotype) and E-cadherin (epithelial phenotype). As a result, T4O treated cSCC cell lines showed prominently decreased N-cadherin expression and upregulated E-cadherin expression (Fig. 4F). Moreover, immunofluorescence confirmed that E-cadherin expression was markedly elevated, while N-cadherin expression was reduced in cells treated with T4O (Fig. 4G). These results suggest that T4O reduces cSCC cell migration and invasive potential by inhibiting EMT.

### T4O inhibits cSCC tumorigenesis in vivo

**Figure 5:**
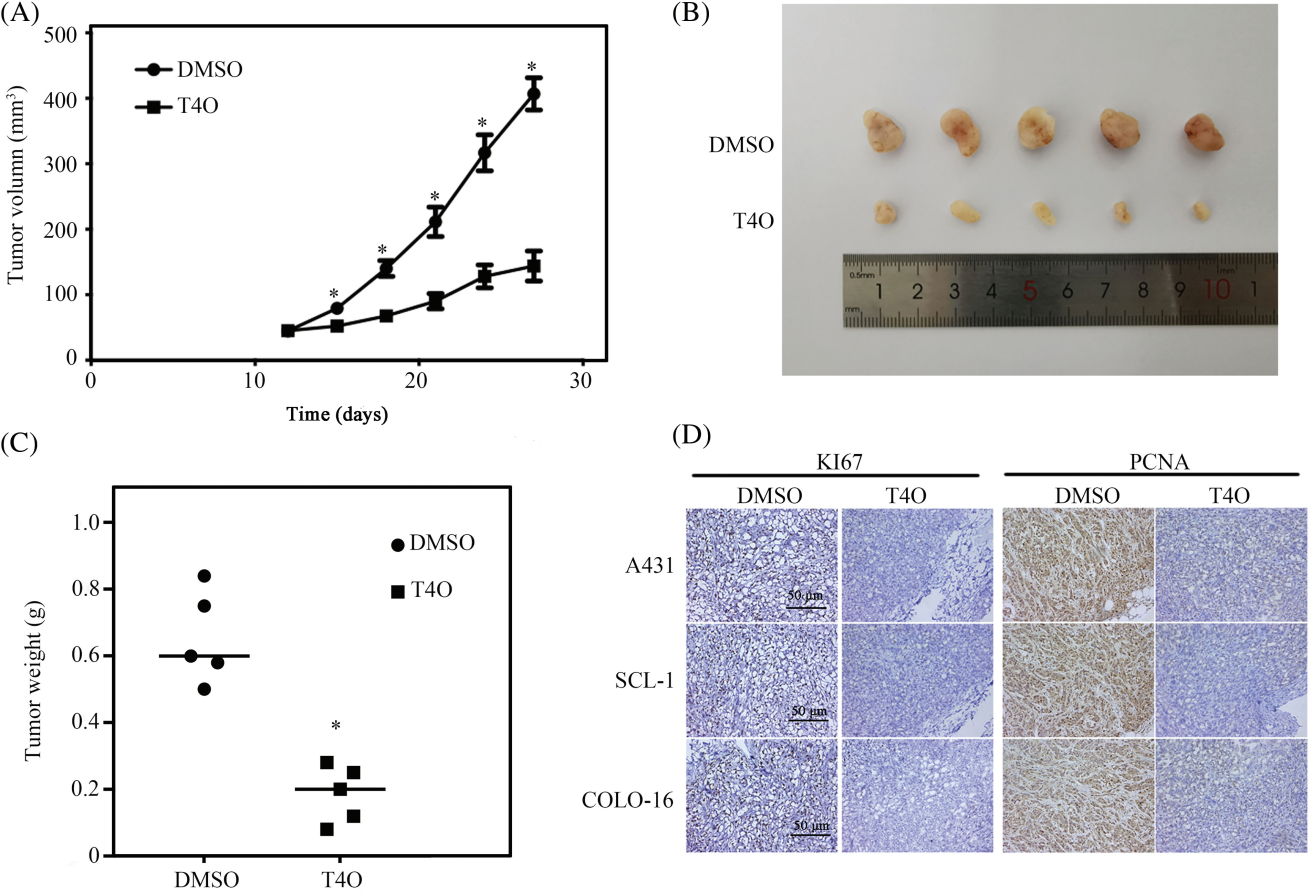
T4O inhibits cSCC tumorigenesis *in vivo*. cSCC tumor xenografts were created in BALB/c nude mice via subcutaneous injection of human A431 cells. Mice were treated with DMSO (control) or T4O and sacrificed 27 days after treatment initiation. (A, B) Tumor volumes at different time points after modeling. (C) Tumor weights. (D) IHC analysis of Ki67 and PCNA expression in tumor samples. *p < 0.05.

To assess the impact of T4O on cSCC cell growth *in vivo*, we established a xenograft tumor model by implanting human A431 cSCC cells into the right axillae of BALB/c nude female mice. Compared to control mice that received DMSO (the vehicle), a significantly reduced tumor burden was recorded in mice treated with T4O (Fig. 5A–C). Consistent with these findings, immunohistochemical analysis of tumor samples revealed a notable decrease in the expression levels of two proliferation markers, Ki67 and PCNA, in T4O-treated mice compared to control animals (Fig. 5D).

### Calpain-2 is a critical mediator of T4O’s anti-tumor activity

**Figure 6:**
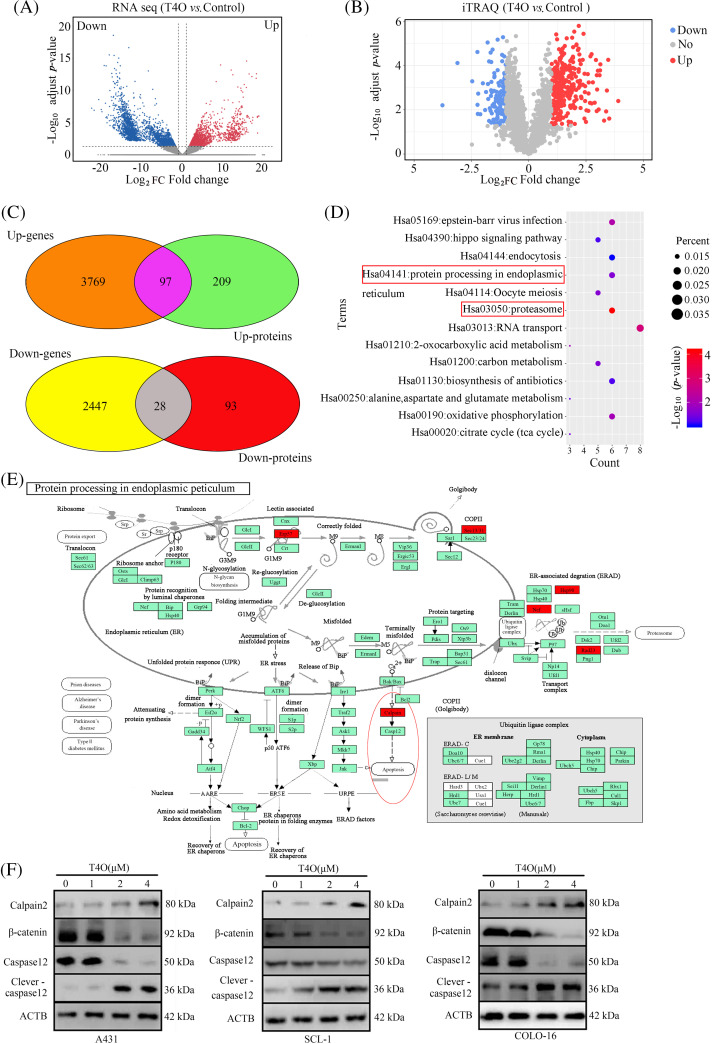
Calpain-2 is a key target of T4O. (A, B) DE genes and proteins were identified in T4O-treated cSCC cells using RNA-sequencing and iTRAQ, respectively. (C) Intersection analysis was performed to analyze genes and proteins with uniform variation trends. (D) KEGG analysis was performed to determine pathway enrichment for differentially expressed genes and proteins. (E) Protein-protein interaction network analysis indicates interaction between calpain-2 and both caspase-12 and β-catenin. (F) Western blotting of calpain-2, β-catenin, caspase-12, and cleaved caspase-12 in cSCC cells treated with T4O.

To elucidate the molecular mechanisms mediating the antitumor actions of T4O on cSCC cells, we sequenced the genome-wide RNA expression of the cells. In total, 2475 downregulated genes and 3866 upregulated genes were identified in T4O-treated cells (Fig. 6A). Subsequently, 306 upregulated proteins and 121 downregulated proteins were identified in T4O-treated cells using the isobaric tag for relative and absolute quantitation (iTRAQ) analysis (Fig. 6B). Then, intersection analysis identified 125 genes and proteins that had a consistent trend of gene expression change following T4O exposure (Fig. 6C).

KEGG enrichment analysis found that these 125 DE genes were significantly enriched in several pathways, including “Epstein-Barr virus infection”, “hippo signaling pathway”, “endocytosis”, “protein processing in endoplasmic reticulum”, “oocyte meiosis”, and “proteasome” (Fig. 6D). Interestingly, calpain-2 was the key node in several of these pathways, including “protein processing in endoplasmic reticulum” and “proteasome” (Fig. 6D).

Protein-protein interaction (PPI) analysis in the STRING database (https://doi.org/10.1093/nar/gkaa1074, accessed on 23 August 2021) showed direct interaction between calpain-2 and key proteins involved in cSCC progression, such as caspase-12 and β-catenin (Fig. 6E). Western blotting showed that calpain-2 expression was significantly elevated in T4O-treated cSCC cells (Fig. 6F). In contrast, T4O treatment significantly decreased β-catenin and caspase-12 expression and increased cleaved caspase-12 expression in cSCC cells. These findings indicate that calpain-2 is a hub-target gene via which T4O inhibits cSCC progression by promoting proteolytic cleavage of β-catenin and caspase-12 (Fig. 6F).

### Calpain-2 silencing suppressed T4O’s inhibitory effect on cSCC cell proliferation and migration

**Figure 7:**
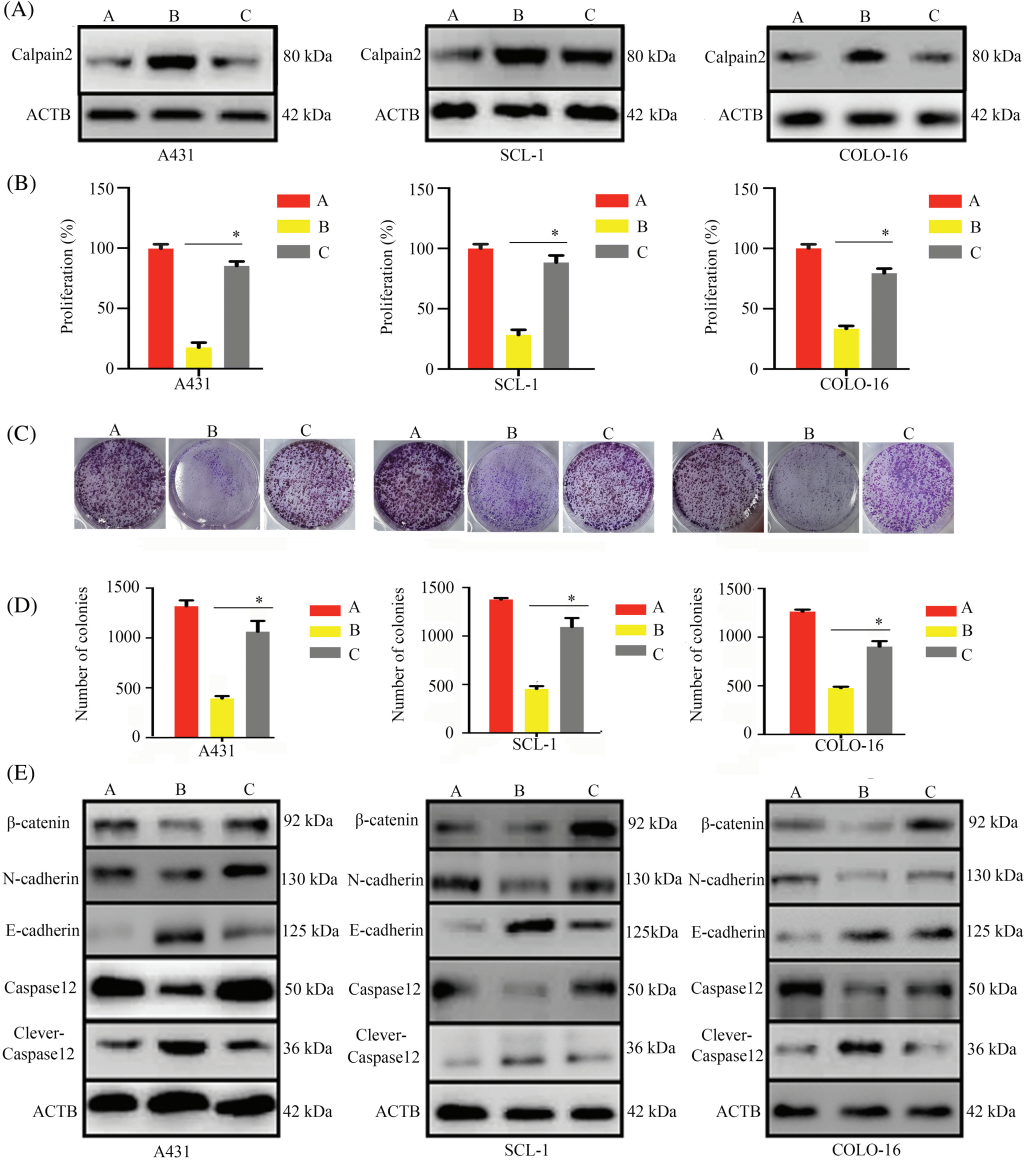
Calpain-2 knockdown reverses the inhibitory effect of T4O on cSCC cell proliferation. cSCC cells were transfected with negative control or calpain-2-targeting siRNAs (si-calpain2) and three experimental groups were defined: negative control siRNA + DMSO, negative control siRNA+ 4 μM T4O, and si-calpain2 + 4 μM T4O. (A) Western blotting was applied to assess the expression of calpain-2 in each experimental group. (B) The effect of calpain-2 silencing on the proliferative rate of cSCC cells was evaluated through CCK-8 assays performed 48 h after DMSO or T4O treatment. (C, D) Colony formation assays were carried out to assess the effect of calpain-2 knockdown on clonogenic survival of cSCC cells treated with different concentrations (0, 1, 2, and 4 μM) of T4O. (E) Western blot analysis of E-cadherin, N-cadherin, β-catenin, caspase-12, and cleaved caspase-12. *p < 0.05.

To explore whether calpain-2 is a central effector gene of the antitumoral actions of T4O, we silenced calpain-2 expression in cSCC cells via transfection of calpain-2-targeting siRNAs (si-calpain2). Western blotting showed that T4O-mediated calpain-2 upregulation was significantly inhibited in calpain-2 silenced cSCC cells (Fig. 7A). In addition, CCK-8 assays showed that calpain-2 knockdown attenuated T4O’s inhibition on cSCC cell proliferation (Fig. 7B) and colony formation capacity (Fig. 7C,D). Moreover, in calpain-2 knockdown cells, the expression of N-cadherin, β-catenin, and caspase-12 was drastically increased, whereas E-cadherin and cleaved caspase-12 levels were reduced in T4O-treated cells (Fig. 7E).

**Figure 8:**
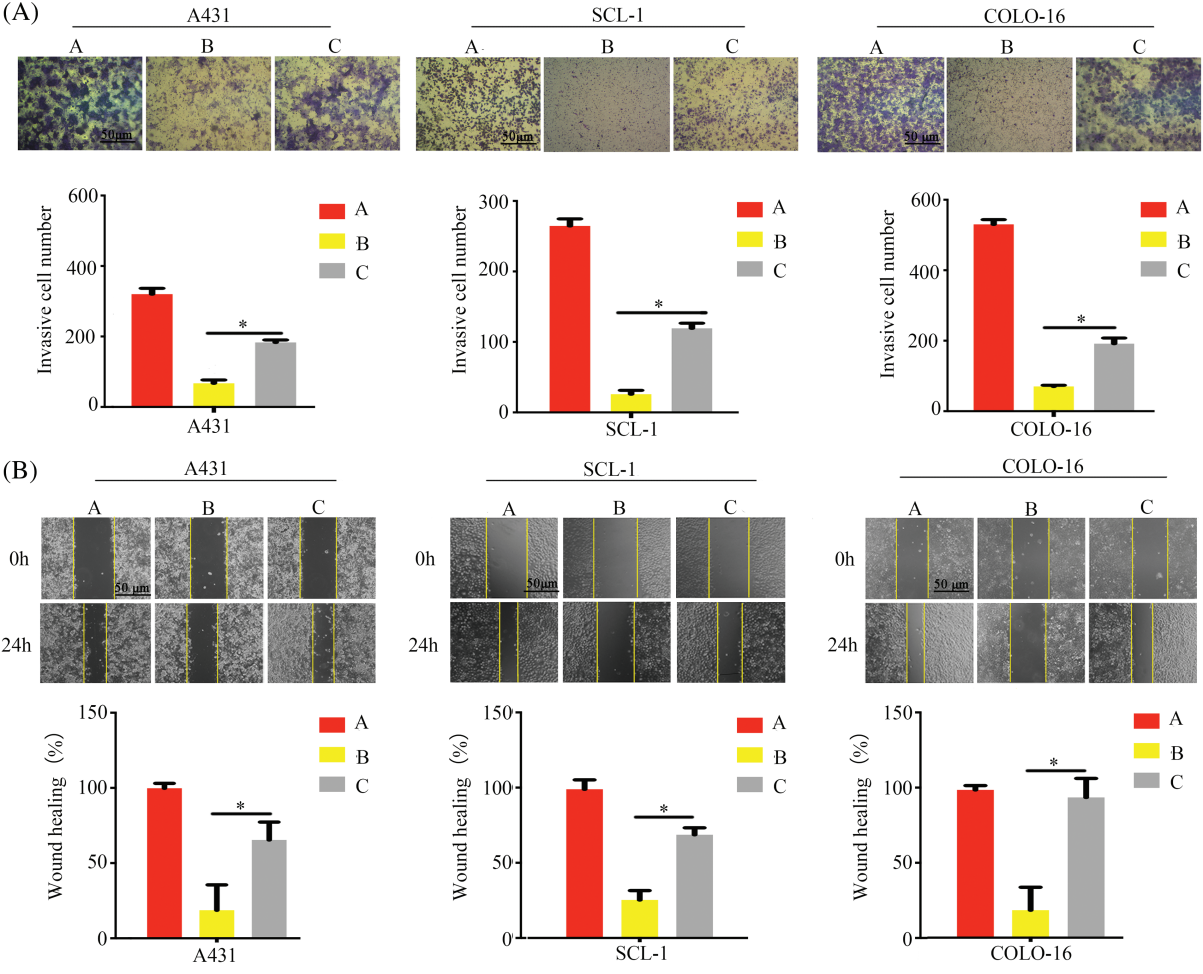
Calpain-2 knockdown abrogates the suppressive effect of T4O on cSCC cell migration and invasion. (A) Matrigel-Transwell invasive ability assessment after calpain-2’s silencing in control and T4O-treated cSCC cells. (B) Wound healing assessment after calpain-2’s silencing in control and T4O-treated cSCC cells. *p < 0.05.

Additionally, wound healing and Transwell invasion assays further demonstrated that calpain-2 knockdown significantly compromised T4O’s inhibitory effect on cSCC cell migration (Fig. 8A) and invasion (Fig. 8B). This evidence indicated that calpain-2 is a key effector gene of T4O-mediated suppression of cSCC cell progression and metastatic potential.

## Discussion

cSCC is a common skin tissue malignancy characterized by high recurrence and metastasis rates and a grave burden on patients’ life quality. Despite surgical excision offering a high cure rate, the challenges of tumor recurrence and metastasis persist [22], underscoring the urgent need to unveil the mechanisms driving cSCC progression and develop novel therapeutic approaches. Increasing evidence has validated many natural products’ antitumor properties and low toxicity and there is marked interest in incorporating natural products into standard therapeutic schedules [23]. Indeed, numerous bioactive molecules derived from medicinal plants are currently used as first-line anticarcinoma agents, including vinca alkaloids (e.g., vinblastine, vincristine) isolated from Vinca rosea, taxanes (e.g., paclitaxel) derived from the Pacific yew bark, and podophyllotoxins (e.g., etoposide) extracted from the root and rhizomes of the Podophyllum species [24].

T4O, a monomeric compound present in many plants’ essential oils, has demonstrated significant antitumor, anti-inflammatory, and antibacterial effects [25]. T4O induces necrotic and apoptosis in murine mesothelioma and melanoma [26,27], and exerts a significant antiproliferative effect in colorectal, pancreatic, prostate, and gastric cancer cells [28]. Further, T4O inhibited ER stress-induced vascular calcification in chronic kidney disease mice by stimulating SIRT1-mediated inhibition of the PERK PERK-eIF2α-ATF4 pathway [29]. In this study, T4O demonstrated a significant inhibition effect on cSCC cell’s proliferation without affecting the growth of normal skin SW1 fibroblasts. Consistent with the above findings, colony formation assays revealed a significant inhibition of clonogenic survival in cSCC cells treated with T4O. Coupled with dysregulated growth, apoptosis resistance is a key factor contributing to tumor progression. Our flow cytometry assays showed that T4O’s inhibition of cSCC proliferation was associated with G1 arrest and induction of apoptosis. Moreover, our experiments using wound healing and Matrigel-Transwell assays revealed T4O’s antimetastatic potential by significantly suppressing cSCC cell migration and invasion. More importantly, consistent with our *in vitro* results, experiments in a mouse xenograft model further demonstrated that T4O significantly inhibits cSCC tumorigenesis *in vivo*. In summary, these findings provide robust evidence of T4O’s antineoplastic effects on cSCC.

The RNA-sequencing and iTRAQ technologies provide powerful tools for exploring molecular mechanisms through gene and protein expression analysis [30]. Applying both techniques, we obtained a total of 125 DE genes and proteins with the same regulation trends, among which we further identified calpain-2 as a T4O’s potential regulatory target. Through PPI network analysis, two proteins closely involved in cSCC progression, namely caspase-12 and β-catenin, were identified as direct targets of calpain-2.

Physiological, environmental, and oncogenic stress activates the calcium-activated Cysteine proteases in the Calpain family [31]. Dysregulated expression of calpain-2 is frequently observed in tumor cells and contributes to tumor suppression or progression in different types of cancers [32–34]. Calpains are involved in cancer cells’ proliferation, migration, and apoptosis regulation in two directions. A slight increase in calpain-2 expression promotes cancer progression through cleaving different tumor suppressors, while significant overexpression of calpain-2 induces apoptosis via cleaving a number of proteins necessary for cell survival [18].

In our xenograft tumor model, we observed increased calpain-2 expression in cSCC cells following T4O treatment. Elevated calpain-2 expression is a common phenomenon observed in malignant cells after treatment with certain antitumoral Substances, and it has been affiliated with enhanced treatment efficacy. A previous study has demonstrated that a calpain-2 inhibitor therapy could reduce colitis-associated cancer in murine models [35]. Additionally, Sapili et al. revealed that geranylated 4-phenylcoumarins promote caspase-independent death of prostate cancer cell lines by targeting calpain-2 and cathepsin B [36]. These studies highlighted that modulation of calpain-2 expression may be a valuable strategy for carcinoma therapy. Dysregulated β-catenin expression, apoptosis resistance, and EMT activation are common features in the etiopathogenesis of many cancer types [37–39]. Our results showed that T4O exposure decreased N-cadherin, β-catenin, and caspase-12 expression and increased cleaved caspase-12 and E-cadherin levels in cultured cSCC cells. These changes were reversed after siRNA-mediated calpain-2 silencing. Furthermore, calpain-2 knockdown significantly reduced T4O’s inhibitory effect on cSCC cell proliferation and metastatic capacity, indicating that calpain-2 is a key mediator of T4O’s antitumor properties. This study offers insights for both research and practice. Specifically, it provides empirical evidence that T4O exerts antitumor effects on cSCC. This model could serve as a foundation for empirical investigations into treatments for other types of carcinomas. However, there are some limitations in this study, such as it lacks a more specific mechanism study. Therefore, future studies could consider additional factors to advance research in skin tissue engineering. While further research is warranted, our findings suggest that T4O treatment may hold promise for enhancing the efficacy of current therapies for recurrent or aggressive cSCC. Additionally, our study underscores the importance of investigating natural compounds like T4O as potential therapeutics for cancer treatment, providing insights for future research and clinical practice.

## Conclusion

In conclusion, this study provides compelling evidence for T4O’s antitumor effects on cSCC via upregulation of calpain-2. The purpose of this study is to find a drug that can effectively treat skin squamous cell carcinoma and provide ideas for future clinical treatment.

## Data Availability

This research’s experimental data are available upon request from the first and corresponding authors.
